# Post-Translational Modification of GPX4 is a Promising Target for Treating Ferroptosis-Related Diseases

**DOI:** 10.3389/fmolb.2022.901565

**Published:** 2022-05-12

**Authors:** Can Cui, Fei Yang, Qian Li

**Affiliations:** ^1^ School of Basic Medical Sciences, Capital Medical University, Beijing, China; ^2^ Advanced Innovation Center for Human Brain Protection, Beijing Key Laboratory of Neural Regeneration and Repair, Capital Medical University, Beijing, China; ^3^ Department of Neurobiology, School of Basic Medical Sciences, Capital Medical University, Beijing, China; ^4^ Department of Biochemistry and Molecular Biology, School of Basic Medical Sciences, Capital Medical University, Beijing, China; ^5^ Beijing Key Laboratory of Cancer Invasion and Metastasis Research, Capital Medical University, Beijing, China

**Keywords:** ferroptosis, post-translational modifications, cancer, enzyme, GPX4

## Abstract

Glutathione peroxidase 4 (GPX4) is one of the most important antioxidant enzymes. As the key regulator of ferroptosis, GPX4 has attracted considerable attention in the fields of cancer, cardiovascular, and neuroscience research in the past 10 years. How to regulate GPX4 activity has become a hot topic nowadays. GPX4 protein level is regulated transcriptionally by transcription factor SP2 or Nrf2. GPX4 activity can be upregulated by supplementing intracellular selenium or glutathione, and also be inhibited by ferroptosis inducers such as ML162 and RSL3. These regulatory mechanisms of GPX4 level/activity have already shown a great potential for treating ferroptosis-related diseases in preclinical studies, especially in cancer cells. Until recently, research show that GPX4 can undergo post-translational modifications (PTMs), such as ubiquitination, succination, phosphorylation, and glycosylation. PTMs of GPX4 affect the protein level/activity of GPX4, indicating that modifying these processes can be a potential therapy for treating ferroptosis-related diseases. This article summarizes the protein characteristics, enzyme properties, and PTMs of GPX4. It also provides a hypothetical idea for treating ferroptosis-related diseases by targeting the PTMs of GPX4.

## Introduction

In 1982, a new glutathione peroxidase was first isolated from the pig liver (Ursini et al., 1982). Unlike previously discovered glutathione peroxidase 1-3 (GPX1-3), this new enzyme, phospholipid hydroperoxide glutathione peroxidase (PHGPX), acts directly on peroxidized phospholipids in the membrane. GPX1-3 are tetramers that mainly reduce H_2_O_2_ and fatty acid hydroperoxide (Gladyshev and Hatfield, 1999; Olson et al., 2010), whereas PHGPX is a monomeric protein and reduces lipid hydroperoxides specifically (Schuckelt et al., 1991). Later, PHGPX was renamed glutathione peroxidase 4 (GPX4) (Nam et al., 1998). Until 2014, GPX4 was identified as the central regulator of ferroptosis, which is an iron-dependent, non-apoptotic, programmed necroptotic cell death form (Dixon et al., 2012). How to regulate the protein level of GPX4 and its activity has become a hot topic to discover the potential treatment of ferroptosis-related diseases recently.

## Enzyme Properties of GPX4 and its Role in Cancer Therapies

**FIGURE 1:**
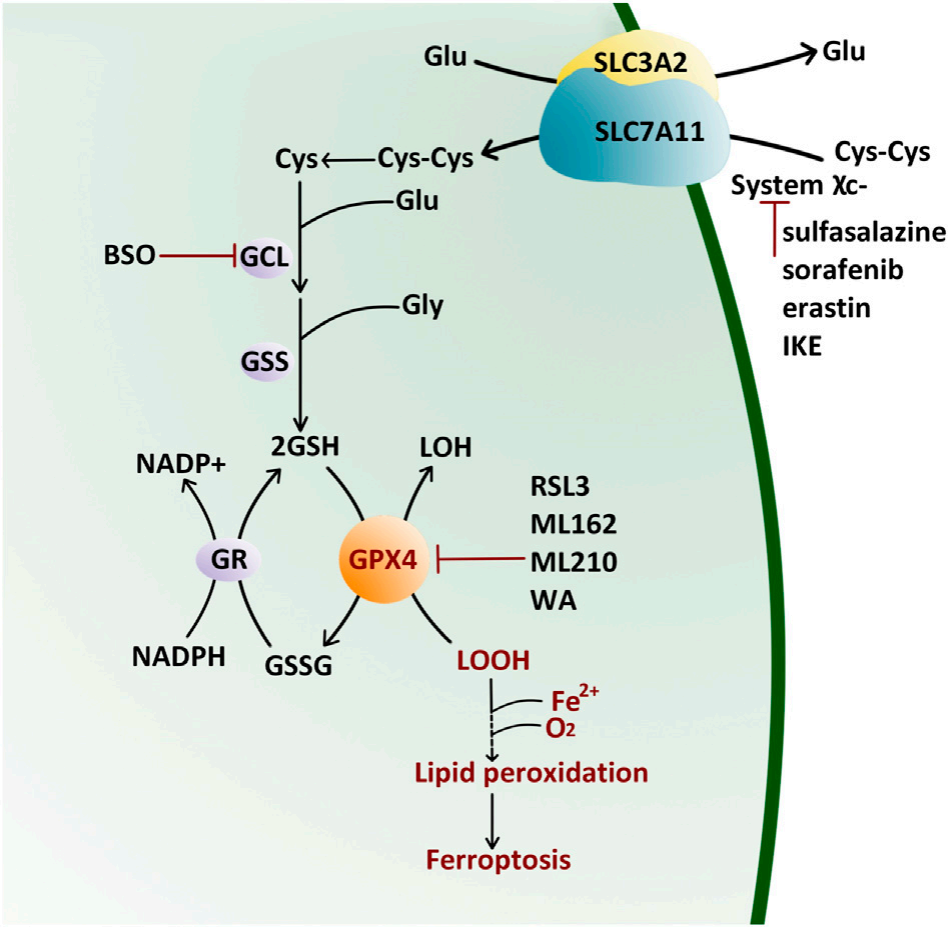
Antioxidant Reaction of GPX4 by the Ping-Pong Pattern. GPX4 reduces lipid hydroxide (LOOH) to alcohol (LOH) and then supplements its active site residues through glutathione (GSH) which is synthesized from cystine (Cys-Cys) pumped by the (system χ_C_^−^). Glu: glutamate; Gly: glycine; GCL: glutamate–cysteine ligase; GSS: glutathione synthetase; BSO: l-buthionine sulfoximine; GR: glutathione reductase; NADP: nicotinamide adenine dinucleotide phosphate; GSSG: oxidized glutathione; IKE: imidazole ketone erastin; RSL3: Ras-selective lethal small molecule three; WA: withaferin A.

The selenoprotein GPX4 has high antioxidant activity. It directly reduces phospholipid hydroperoxide through a conserved catalytic triad of selenocysteine 46, asparagine 81, and tryptophane 136 (Maiorino et al., 1995). The reaction kinetics of GPX4 is described as a Ping-Pong pattern that is embodied in two phases (Takebe et al., 2002) (Figure 1). Firstly, the peroxide is reduced through the active site selenocysteine 46 and then converted to the oxidized form (selenenic acid derivative). The second stage is to recharge the oxidized catalytic site via glutathione (GSH) (Takebe et al., 2002). The content of GSH and nicotinamide adenine dinucleotide phosphate (NADPH) directly affect the GPX4 activity.

Cystine/glutamate transporter (system χ_C_^−^) on the cell membrane, composed of a catalytic subunit SLC7A11 and a chaperone subunit SLC3A2, pumps cystine from extracellular into cells for GSH biosynthesis (Bannai, 1986; Sato et al., 1999). Therefore, classic ferroptosis inducers (FINs) are divided into two classes (Figure 1): class Ⅰ FIN such as sorafenib (Louandre et al., 2013), sulfasalazine (Gout et al., 2001), erastin (Dixon et al., 2012) and its analog imidazole ketone erastin (IKE) (Zhang et al., 2019), can block the GSH synthesis through inhibiting system χ_C_^−^, and then reduce the GPX4 enzyme activity by GSH depletion; class Ⅱ FIN such as Ras-selective lethal small molecule 3 (RSL3) (Yang and Stockwell, 2008), ML162 (Weïwer et al., 2012), and ML210 (Eaton et al., 2020), can directly inhibit GPX4 activity. They eventually lead to the accumulation of lipid peroxides without the reduction of GSH (Yang et al., 2014). Apart from these two types of FINs, other methods or compounds can also induce ferroptosis (Figure 1): L-buthionine sulfoximine (BSO) suppresses glutamate-cycteine ligase (GCL) and induces GSH depletion (Seiler et al., 2008; Sun et al., 2018), driving cells to ferroptosis; Cystine starvation leads to glutamate accumulation, GSH biosynthesis reduction, and GPX4 translation blockage, therefore sensitizing cancer cells to ferroptosis (Zhang Y. et al., 2021). FINs and other compounds that cause ferroptosis drive tumor cell death, sensitizing cancer cells to radiotherapy and chemotherapy in prostate cancer (Li et al., 2021), colorectal cancer (Sui et al., 2018), osteosarcoma (Liu and Wang, 2019), and leukemia (Yu et al., 2015). Therefore, traditional chemotherapy/radiotherapy combined with FINs treatment is beneficial to reverse multidrug resistance.

## Post-Translational Modifications of GPX4 in Cancer Cells

**FIGURE 2:**
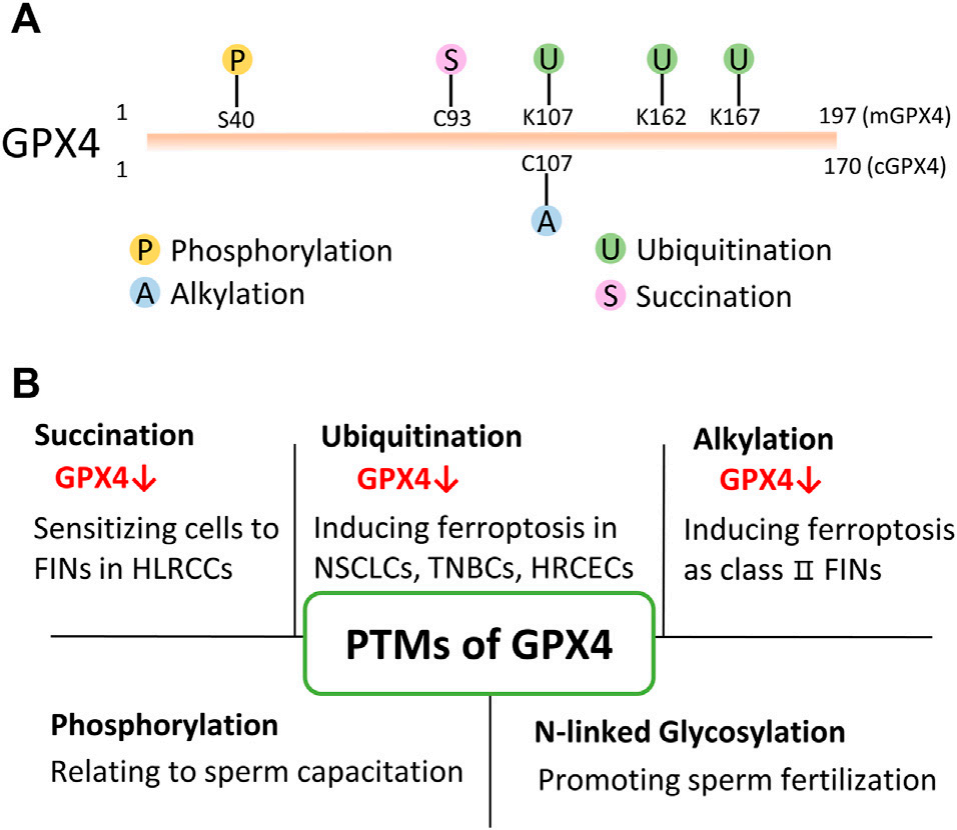
PTMs of GPX4. **(A)** Present identified PTMs sites of GPX4 from proteomics and drug research. mGPX4: mitochondrial isoform; cGPX4: cytosolic isoform. **(B)** Various PTMs have been found to modify GPX4 protein and regulate its protein level/activity in physiological and/or pathophysiological conditions. HLRCC: hereditary leiomyomatosis and renal cell cancer; NSCLS: non-small cell lung cancer; TNBC: triple-negative breast cancer cell; HRCEC: human retinal capillary endothelial cell; FINs: ferroptosis inducers.

Besides small molecular compounds like FINs, post-translational modifications (PTMs) also provide an emerging perspective to regulate its protein level/activity (Figure 2). Thus, studying and targeting PTMs of GPX4 has a great potential to treat cancers in the future.

### Succination of GPX4

Succination was discovered in 2006 as a non-enzymatic, irreversible protein modification (Alderson et al., 2006). This post-translational modification is mediated by fumarate. Fumarate is an intermediate of the Krebs cycle in mitochondria. It binds to the sulfhydryl group of the cysteine residue and forms a thioether bond in the absence of an enzyme (Blatnik et al., 2008; Manuel and Frizzell, 2013). An elegant study showed that intracellular fumarate aggregation led to the succination of GPX4 at cysteine 93 (mono- and di-succination), which significantly reduced the enzymatic activity of GPX4 and sensitized cancer cells to FINs (Kerins et al., 2018). In addition, fumarate hydratase (FH)-inactivated cells were shown to be synthetic lethal with FINs (Kerins et al., 2018). This study suggests that targeting PTMs of GPX4 is a promising therapeutic strategy for ferroptosis-related diseases for the first time.

### Ubiquitination of GPX4

Ubiquitination is a reversible process in which the ubiquitin is added to lysine residue(s) of the substrate protein by three different types of enzymes, E1, E2, and E3. The process of ubiquitination ultimately renders proteins to degradation by the proteasome thereby affecting intracellular protein levels (Popovic et al., 2014). On the other hand, deubiquitinases (DUBs) remove ubiquitin chains from ubiquitinated proteins (Ronau et al., 2016). E3 ligase and DUB are both having substrate specificity.

Proteomic analyses reveal that GPX4 could be ubiquitinated at lysine 107, 162, and 167 (Wagner et al., 2011; Akimov et al., 2018). However, those hypothetically ubiquitinated residues have not been verified in bench works. Whereas, a study showed that when treating non-small cell lung cancer cells with the DUB inhibitor, pyridinium sulfur palladium complex (PdPT), GPX4 was ubiquitinated and degraded (Yang et al., 2020). Subsequently, cancer cell growth was restrained by inducing GPX4-dependent ferroptosis (Yang et al., 2020). Later, another study showed that a Parthenolide (PTL)-derived drug DMOCPTL directly bound to the GPX4 active site (selenocysteine 46), and led to GPX4 ubiquitination in triple-negative breast cancer cells (Ding et al., 2021). Unfortunately, the above-mentioned studies did not reveal the ubiquitinated residue(s) of GPX4.

Discovering the E3 ligase is also very important for understanding the regulatory mechanisms of protein ubiquitination. A very recent study discovered that the E3 motif-containing 46 (TRIM46) might be one of the E3 ligases of GPX4 in human retinal capillary endothelial cells with high glucose treatment (Zhang J. et al., 2021). Whether GPX4 has other E3 ligases is needed further investigation. These findings indicate that GPX4 can be ubiquitinated under certain circumstances. Importantly, treating cells with PdPT, DMOCPTL, or other similar compounds could potentially kill cancer cells by inducing ferroptosis.

### Alkylation of GPX4

Alkylation is a chemical process of introducing one or more alkyl groups into a protein or a compound. An alkyl group is an alkane molecule that is missing a hydrogen atom. For example, methyl groups are the simplest alkyl and are added to various proteins including core histones (Chen et al., 1999; Strahl et al., 1999). Previous studies have shown several class Ⅱ FINs such as RSL3 (Yang and Stockwell, 2008) and ML162 (Weïwer et al., 2012) exerted covalent inhibition activity. These class Ⅱ FINs bound to selenocysteine 46 residue of GPX4 via an electrophilic alkyl chloride moiety, mediating alkylation on GPX4 (Eaton et al., 2020; Vučković et al., 2020). Besides selenocysteine 46, GPX4 contains several cysteine residues which are also nucleophilic sites. A report showed that GPX4 was most likely alkylated at cysteine 107 with the addition of withaferin A (Grossman et al., 2017). In addition, the alkylation of GPX4 resulted in ferroptosis in high-risk neuroblastoma (Hassannia et al., 2017). However, Hassaniia *et al* did not further investigate the specifically modified residue(s) of GPX4 or which kind(s) of alkylation occurs on GPX4.

## Post-Translational Modifications of GPX4 in Other Conditions

### Phosphorylation of GPX4 is Involved in Sperm Maturation

Phosphorylation is one of the most common PTMs involved in the regulation of protein stability and activity. It occurs on serine, tyrosine, and threonine residues of target proteins and is regulated by protein kinases and phosphatases (Olsen et al., 2006). Phosphorylation of GPX4 has been found in the pig heart (Brigelius-Flohé et al., 1994), golden hamster’s sperms (Nagdas et al., 2005), and rat testicles (Lundby et al., 2012). Notably, the mature testicle is the organ with the highest GPX4 activity in mammals (Vernet et al., 2004), and sperm express high levels of GPX4 (Nam et al., 1998). Studies showed that GPX4 lost its enzyme activity during the differentiation and maturation of sperm (Ursini et al., 1999). Meanwhile, the phosphorylation of GPX4 on tyrosine has been found in capacitated sperm of the golden hamster (Nagdas et al., 2005). Several proteomic analyses also revealed that human GPX4 might be phosphorylated at serine 40 (Mertins et al., 2014; Mertins et al., 2016). These data indicate that phosphorylation of GPX4 participates in the GPX4 activity regulation. However, the exact phosphorylated residues, the corresponding phosphatases, and the biological effects of phosphorylation on GPX4 need to be further investigated.

### N-Linked Glycosylation of GPX4 is Related to the Fertilization of Sperm

Glycosylation (N-linked and O-linked) is under the reaction of glycosyltransferases (Reily et al., 2019). Protein glycosylation affects protein stability and folding (Molinari, 2007) and leads to changes in protein function. GPX4 has been identified as a membrane glycoprotein in an N-linked glycoproteome study in human sperm. The modification site was at the asparagine residue of GPX4 (Wang et al., 2013). In addition, deglycosylation treatment was also found to significantly decrease sperm fertilization rate (Wang et al., 2013). This study suggests that N-linked glycosylation plays an important role in modifying membrane GPX4 activity and enables sperm to exert their normal fertilization functions.

### SUMOylation of GPX4 Might Mediate Lipid Peroxidation Inhibition

In addition to phosphorylation and glycosylation, SUMOylation is also predicted to occur on GPX4. SUMOylation is a small ubiquitin-like modifier (SUMO) connected to the substrate protein through a tertiary enzymatic reaction (Tatham et al., 2001). It often modifies lysine residues of substrate proteins (Tatham et al., 2001). SUMOylation regulates the protein-protein interactions, mediates the function and localization of substrate proteins, and increases protein stability (Ouyang et al., 2009; Li et al., 2016; Han et al., 2018). A study using software resources (SUMOplot) for bioinformatics analysis predicted that the residues of GPX4 most likely to be modified by SUMO were lysine 74, 106, and 125 (Sheng et al., 2021). The cationic region of GPX4, including lysine 125, has the potential to interact with the phospholipid bilayer. Then the redox-active center of GPX4 was directed to the H_2_O_2_ group in the phospholipid fatty acid chain, resulting in the inhibition of lipid peroxidation of the cell membrane in silico (Cozza et al., 2017; Sheng et al., 2021).

Since GPX4 is a core regulator of ferroptosis, although it is unknown whether the phosphorylation, glycosylation or SUMOylation of GPX4 affect ferroptosis, it would be of interest to investigate it in future translational studies.

## Discussion and Future Direction

PTMs are an important regulator of protein level and activity with huge potential in translational and clinical research. For instance, S-farnesylation is one of the post-translational protein lipid modifications (Nagase et al., 1997; Berndt et al., 2011). Hutchinson–Gilford progeria syndrome (HGPS) is a genetic rare disease caused by progerin formation due to a deficiency of removing a farnesylcysteine-containing peptide from prelamin A (Eriksson et al., 2003). Consequently, prelamin A turned into farnesylated and non-functional progerin instead of lamin A (Young et al., 2005). However, progein was identified to be the critical target of farnesyltransferase inhibitors (FTIs) (Yang et al., 2010) and one FTI called lonafarnib have been tested in a cohort study which showed a significantly lower mortality rate (hazard ratio, 0.12) after a median of 2.2 years of follow-up (Gordon et al., 2018). S-palmitoylation is also an important PTM in many oncoproteins such as RAS-family GTPases (Dekker et al., 2010) and epidermal growth factor receptor (EGFR) and tumor suppressors such as melanocortin one receptor (MRC1) (Chen et al., 2017), which serves as an important target pathway to inhibit tumor growth and prevent tumorigenesis (Hernandez et al., 2017). Inducing palmitoylation in MCR1variant individuals can lower their risk to develop melanoma (Chen et al., 2017). Targeting inhibition of palmitoylation has also shown sensitization to gefitinib in cancer cells (Bollu et al., 2015).

The investigation of the GPX4 function was far ahead of the emergence of ferroptosis. A variety of PTMs of GPX4 has been observed in both physiological and pathophysiological conditions. Targeting PTMs of GPX4 could be a promising strategy for treating diseases, including ferroptosis-related diseases such as cancers, neurodegenerative disorders, and ischemic-reperfusion diseases as well as other important players in ferroptosis (Wei et al., 2020). However, in contrast to other proteins, such as TP53 (Liu et al., 2019), studies on PTMs of GPX4 have only just started. At this point, we believe it is important to explore several aspects in future studies, such as to discover whether some of the PTMs summarized above affect GPX4 activity in bench works and GPX4 dysfunction-relative diseases. What are the corresponding enzymes of the above-mentioned PTMs? If other PTMs, such as methylation, lipoylation, or newly identified lactation also modify GPX4 protein? Since ferroptosis is an important cell death form in various conditions, and GPX4 is its core regulator, more in-depth studies are urgently needed to assist GPX4-based clinical therapeutic strategies for tumor killing and neuronal protection.

## References

[B1] Akimov V. Barrio-Hernandez I. Hansen S. V. F. Hallenborg P. Pedersen A.-K. Bekker-Jensen D. B. (2018). UbiSite Approach for Comprehensive Mapping of Lysine and N-Terminal Ubiquitination Sites. Nat. Struct. Mol. Biol. 25 (7), 631–640. 10.1038/s41594-018-0084-y 29967540

[B2] Alderson N. L. Wang Y. Blatnik M. Frizzell N. Walla M. D. Lyons T. J. (2006). S-(2-Succinyl)cysteine: a Novel Chemical Modification of Tissue Proteins by a Krebs Cycle Intermediate. Archives Biochem. Biophysics 450 (1), 1–8. 10.1016/j.abb.2006.03.005 16624247

[B3] Bannai S. (1986). Exchange of Cystine and Glutamate across Plasma Membrane of Human Fibroblasts. J. Biol. Chem. 261 (5), 2256–2263. 10.1016/s0021-9258(17)35926-4 2868011

[B4] Berndt N. Hamilton A. D. Sebti S. M. (2011). Targeting Protein Prenylation for Cancer Therapy. Nat. Rev. Cancer 11 (11), 775–791. 10.1038/nrc3151 22020205 PMC4037130

[B5] Blatnik M. Thorpe S. R. Baynes J. W. (2008). Succination of Proteins by Fumarate. Ann. N. Y. Acad. Sci. 1126, 272–275. 10.1196/annals.1433.047 18448829 PMC2423376

[B6] Bollu L. R. Katreddy R. R. Blessing A. M. Pham N. Zheng B. Wu X. (2015). Intracellular Activation of EGFR by Fatty Acid Synthase Dependent Palmitoylation. Oncotarget 6 (33), 34992–35003. 10.18632/oncotarget.5252 26378037 PMC4741504

[B7] Brigelius-Flohé R. Aumann K. D. Blöcker H. Gross G. Kiess M. Klöppel K. D. (1994). Phospholipid-hydroperoxide Glutathione Peroxidase. Genomic DNA, cDNA, and Deduced Amino Acid Sequence. J. Biol. Chem. 269 (10), 7342–7348. 8125951

[B8] Chen D. Ma H. Hong H. Koh S. S. Huang S.-M. Schurter B. T. (1999). Regulation of Transcription by a Protein Methyltransferase. Science 284 (5423), 2174–2177. 10.1126/science.284.5423.2174 10381882

[B9] Chen S. Zhu B. Yin C. Liu W. Han C. Chen B. (2017). Palmitoylation-dependent Activation of MC1R Prevents Melanomagenesis. Nature 549 (7672), 399–403. 10.1038/nature23887 28869973 PMC5902815

[B10] Cozza G. Rossetto M. Bosello-Travain V. Maiorino M. Roveri A. Toppo S. (2017). Glutathione Peroxidase 4-catalyzed Reduction of Lipid Hydroperoxides in Membranes: The Polar Head of Membrane Phospholipids Binds the Enzyme and Addresses the Fatty Acid Hydroperoxide Group toward the Redox Center. Free Radic. Biol. Med. 112, 1–11. 10.1016/j.freeradbiomed.2017.07.010 28709976

[B11] Dekker F. J. Rocks O. Vartak N. Menninger S. Hedberg C. Balamurugan R. (2010). Small-molecule Inhibition of APT1 Affects Ras Localization and Signaling. Nat. Chem. Biol. 6 (6), 449–456. 10.1038/nchembio.362 20418879

[B12] Ding Y. Chen X. Liu C. Ge W. Wang Q. Hao X. (2021). Identification of a Small Molecule as Inducer of Ferroptosis and Apoptosis through Ubiquitination of GPX4 in Triple Negative Breast Cancer Cells. J. Hematol. Oncol. 14 (1), 19. 10.1186/s13045-020-01016-8 33472669 PMC7816340

[B13] Dixon S. J. Lemberg K. M. Lamprecht M. R. Skouta R. Zaitsev E. M. Gleason C. E. (2012). Ferroptosis: an Iron-dependent Form of Nonapoptotic Cell Death. Cell 149 (5), 1060–1072. 10.1016/j.cell.2012.03.042 22632970 PMC3367386

[B14] Eaton J. K. Furst L. Ruberto R. A. Moosmayer D. Hilpmann A. Ryan M. J. (2020). Selective Covalent Targeting of GPX4 Using Masked Nitrile-Oxide Electrophiles. Nat. Chem. Biol. 16 (5), 497–506. 10.1038/s41589-020-0501-5 32231343 PMC7251976

[B15] Eriksson M. Brown W. T. Gordon L. B. Glynn M. W. Singer J. Scott L. (2003). Recurrent De Novo Point Mutations in Lamin A Cause Hutchinson-Gilford Progeria Syndrome. Nature 423 (6937), 293–298. 10.1038/nature01629 12714972 PMC10540076

[B16] Gladyshev V. N. Hatfield D. L. (1999). Selenocysteine-containing Proteins in Mammals. J. Biomed. Sci. 6 (3), 151–160. 10.1007/bf02255899 10343164

[B17] Gordon L. B. Shappell H. Massaro J. D’Agostino R. B. Brazier. Campbell S. E. (2018). Association of Lonafarnib Treatment vs No Treatment with Mortality Rate in Patients with Hutchinson-Gilford Progeria Syndrome. Jama 319 (16), 1687–1695. 10.1001/jama.2018.3264 29710166 PMC5933395

[B18] Gout P. Buckley A. Simms C. Bruchovsky N. (2001). Sulfasalazine, a Potent Suppressor of Lymphoma Growth by Inhibition of the Xc − Cystine Transporter: a New Action for an Old Drug. Leukemia 15 (10), 1633–1640. 10.1038/sj.leu.2402238 11587223

[B19] Grossman E. A. Ward C. C. Spradlin J. N. Bateman L. A. Huffman T. R. Miyamoto D. K. (2017). Covalent Ligand Discovery against Druggable Hotspots Targeted by Anti-cancer Natural Products. Cell Chem. Biol. 24 (11), 1368–1376. 10.1016/j.chembiol.2017.08.013 28919038 PMC5693770

[B20] Han Z.-J. Feng Y.-H. Gu B.-H. Li Y.-M. Chen H. (2018). The Post-translational Modification, SUMOylation, and Cancer (Review). Int. J. Oncol. 52 (4), 1081–1094. 10.3892/ijo.2018.4280 29484374 PMC5843405

[B69] Hassannia B. Wiernicki B. Ingold I. Qu F. Van Herck S. Tyurina Y. Y. (2018). Nano-Targeted Induction of Dual Ferroptotic Mechanisms Eradicates High-Risk Neuroblastoma. J. Clin. Invest 128 (8), 3341–3355. 10.1172/jci99032 29939160 PMC6063467

[B21] Hernandez J. L. Davda D. Cheung See Kit M. Majmudar J. D. Won S. J. Gang M. (2017). APT2 Inhibition Restores Scribble Localization and S -Palmitoylation in Snail-Transformed Cells. Cell Chem. Biol. 24 (1), 87–97. 10.1016/j.chembiol.2016.12.007 28065656 PMC5362123

[B22] Kerins M. J. Milligan J. Wohlschlegel J. A. Ooi A. (2018). Fumarate Hydratase Inactivation in Hereditary Leiomyomatosis and Renal Cell Cancer Is Synthetic Lethal with Ferroptosis Induction. Cancer Sci. 109 (9), 2757–2766. 10.1111/cas.13701 29917289 PMC6125459

[B23] Li M. Chen X. Wang X. Wei X. Wang D. Liu X. (2021). RSL3 Enhances the Antitumor Effect of Cisplatin on Prostate Cancer Cells via Causing Glycolysis Dysfunction. Biochem. Pharmacol. 192, 114741. 10.1016/j.bcp.2021.114741 34428443

[B24] Li S. Wang M. Qu X. Xu Z. Yang Y. Su Q. (2016). SUMOylation of PES1 Upregulates its Stability and Function via Inhibiting its Ubiquitination. Oncotarget 7 (31), 50522–50534. 10.18632/oncotarget.10494 27409667 PMC5226600

[B25] Liu Q. Wang K. (2019). The Induction of Ferroptosis by Impairing STAT3/Nrf2/GPx4 Signaling Enhances the Sensitivity of Osteosarcoma Cells to Cisplatin. Cell Biol. Int. 43 (11), 1245–1256. 10.1002/cbin.11121 30811078

[B26] Liu Y. Tavana O. Gu W. (2019). p53 Modifications: Exquisite Decorations of the Powerful Guardian. J. Mol. Cell Biol. 11 (7), 564–577. 10.1093/jmcb/mjz060 31282934 PMC6736412

[B27] Louandre C. Ezzoukhry Z. Godin C. Barbare J.-C. Mazière J.-C. Chauffert B. (2013). Iron-dependent Cell Death of Hepatocellular Carcinoma Cells Exposed to Sorafenib. Int. J. Cancer 133 (7), 1732–1742. 10.1002/ijc.28159 23505071

[B28] Lundby A. Secher A. Lage K. Nordsborg N. B. Dmytriyev A. Lundby C. (2012). Quantitative Maps of Protein Phosphorylation Sites across 14 Different Rat Organs and Tissues. Nat. Commun. 3, 876. 10.1038/ncomms1871 22673903 PMC3621391

[B29] Maiorino M. Aumann K.-D. Brigelius-Flohé R. Doria D. van den Heuvel J. McCarthy J. (1995). Probing the Presumed Catalytic Triad of Selenium-Containing Peroxidases by Mutational Analysis of Phospholipid Hydroperoxide Glutathione Peroxidase (PHGPx). Biol. Chem. Hoppe-Seyler 376 (11), 651–660. 10.1515/bchm3.1995.376.11.651 8962674

[B30] Manuel A. M. Frizzell N. (2013). Adipocyte Protein Modification by Krebs Cycle Intermediates and Fumarate Ester-Derived Succination. Amino Acids 45 (5), 1243–1247. 10.1007/s00726-013-1568-z 23892396

[B31] Mertins P. Mani D. R. Mani D. R. Ruggles K. V. Gillette M. A. Clauser K. R. (2016). Proteogenomics Connects Somatic Mutations to Signalling in Breast Cancer. Nature 534 (7605), 55–62. 10.1038/nature18003 27251275 PMC5102256

[B32] Mertins P. Yang F. Liu T. Mani D. R. Petyuk V. A. Gillette M. A. (2014). Ischemia in Tumors Induces Early and Sustained Phosphorylation Changes in Stress Kinase Pathways but Does Not Affect Global Protein Levels. Mol. Cell. Proteomics 13 (7), 1690–1704. 10.1074/mcp.M113.036392 24719451 PMC4083109

[B33] Molinari M. (2007). N-glycan Structure Dictates Extension of Protein Folding or Onset of Disposal. Nat. Chem. Biol. 3 (6), 313–320. 10.1038/nchembio880 17510649

[B34] Nagase T. Kawata S. Tamura S. Matsuda Y. Inui Y. Yamasaki E. (1997). Manumycin and Gliotoxin Derivative KT7595 Block Ras Farnesylation and Cell Growth but Do Not Disturb Lamin Farnesylation and Localization in Human Tumour Cells. Br. J. Cancer 76 (8), 1001–1010. 10.1038/bjc.1997.499 9376258 PMC2228099

[B35] Nagdas S. K. Winfrey V. P. Olson G. E. (2005). Tyrosine Phosphorylation Generates Multiple Isoforms of the Mitochondrial Capsule Protein, Phospholipid Hydroperoxide Glutathione Peroxidase (PHGPx), during Hamster Sperm Capacitation1. Biol. Reprod. 72 (1), 164–171. 10.1095/biolreprod.104.033530 15385412

[B36] Nam S.-Y. Fujisawa M. Kim J.-S. Kurohmaru M. Hayashi Y. (1998). Expression Pattern of Phospholipid Hydroperoxide Glutathione Peroxidase Messenger Ribonucleic Acid in Mouse Testis1. Biol. Reprod. 58 (5), 1272–1276. 10.1095/biolreprod58.5.1272 9603263

[B37] Olsen J. V. Blagoev B. Gnad F. Macek B. Kumar C. Mortensen P. (2006). Global, *In Vivo*, and Site-specific Phosphorylation Dynamics in Signaling Networks. Cell 127 (3), 635–648. 10.1016/j.cell.2006.09.026 17081983

[B38] Olson G. E. Whitin J. C. Hill K. E. Winfrey V. P. Motley A. K. Austin L. M. (2010). Extracellular Glutathione Peroxidase (Gpx3) Binds Specifically to Basement Membranes of Mouse Renal Cortex Tubule Cells. Am. J. Physiology-Renal Physiology 298 (5), F1244–F1253. 10.1152/ajprenal.00662.2009 PMC2867408 20015939

[B39] Ouyang K. J. Woo L. L. Zhu J. Huo D. Matunis M. J. Ellis N. A. (2009). SUMO Modification Regulates BLM and RAD51 Interaction at Damaged Replication Forks. PLoS Biol. 7 (12), e1000252. 10.1371/journal.pbio.1000252 19956565 PMC2779653

[B40] Popovic D. Vucic D. Dikic I. (2014). Ubiquitination in Disease Pathogenesis and Treatment. Nat. Med. 20 (11), 1242–1253. 10.1038/nm.3739 25375928

[B41] Reily C. Stewart T. J. Renfrow M. B. Novak J. (2019). Glycosylation in Health and Disease. Nat. Rev. Nephrol. 15 (6), 346–366. 10.1038/s41581-019-0129-4 30858582 PMC6590709

[B42] Ronau J. A. Beckmann J. F. Hochstrasser M. (2016). Substrate Specificity of the Ubiquitin and Ubl Proteases. Cell Res. 26 (4), 441–456. 10.1038/cr.2016.38 27012468 PMC4822132

[B43] Sato H. Tamba M. Ishii T. Bannai S. (1999). Cloning and Expression of a Plasma Membrane Cystine/glutamate Exchange Transporter Composed of Two Distinct Proteins. J. Biol. Chem. 274 (17), 11455–11458. 10.1074/jbc.274.17.11455 10206947

[B44] Schuckelt R. Brigelius-Flohé R. Maiorino M. Roveri A. Reumkens J. Strabburger W. (1991). Phospholipid Hydroperoxide Glutathione Peroxidase Is a Seleno-Enzyme Distinct from the Classical Glutathione Peroxidase as Evident from Cdna and Amino Acid Sequencing. Free Radic. Res. Commun. 14 (5-6), 343–361. 10.3109/10715769109093424 1778506

[B45] Seiler A. Schneider M. Förster H. Roth S. Wirth E. K. Culmsee C. (2008). Glutathione Peroxidase 4 Senses and Translates Oxidative Stress into 12/15-lipoxygenase Dependent- and AIF-Mediated Cell Death. Cell Metab. 8 (3), 237–248. 10.1016/j.cmet.2008.07.005 18762024

[B46] Sheng Z. Zhu J. Deng Y.-n. Gao S. Liang S. (2021). SUMOylation Modification-Mediated Cell Death. Open Biol. 11 (7), 210050. 10.1098/rsob.210050 34255975 PMC8277462

[B47] Strahl B. D. Ohba R. Cook R. G. Allis C. D. (1999). Methylation of Histone H3 at Lysine 4 Is Highly Conserved and Correlates with Transcriptionally Active Nuclei in Tetrahymena. Proc. Natl. Acad. Sci. U.S.A. 96 (26), 14967–14972. 10.1073/pnas.96.26.14967 10611321 PMC24756

[B48] Sui X. Zhang R. Liu S. Duan T. Zhai L. Zhang M. (2018). RSL3 Drives Ferroptosis through GPX4 Inactivation and ROS Production in Colorectal Cancer. Front. Pharmacol. 9, 1371. 10.3389/fphar.2018.01371 30524291 PMC6262051

[B49] Sun Y. Zheng Y. Wang C. Liu Y. (2018). Glutathione Depletion Induces Ferroptosis, Autophagy, and Premature Cell Senescence in Retinal Pigment Epithelial Cells. Cell Death Dis. 9 (7), 753. 10.1038/s41419-018-0794-4 29988039 PMC6037763

[B50] Takebe G. Yarimizu J. Saito Y. Hayashi T. Nakamura H. Yodoi J. (2002). A Comparative Study on the Hydroperoxide and Thiol Specificity of the Glutathione Peroxidase Family and Selenoprotein P. J. Biol. Chem. 277 (43), 41254–41258. 10.1074/jbc.M202773200 12185074

[B51] Tatham M. H. Jaffray E. Vaughan O. A. Desterro J. M. P. Botting C. H. Naismith J. H. (2001). Polymeric Chains of SUMO-2 and SUMO-3 Are Conjugated to Protein Substrates by SAE1/SAE2 and Ubc9. J. Biol. Chem. 276 (38), 35368–35374. 10.1074/jbc.M104214200 11451954

[B52] Ursini F. Heim S. Kiess M. Maiorino M. Roveri A. Wissing J. (1999). Dual Function of the Selenoprotein PHGPx during Sperm Maturation. Science 285 (5432), 1393–1396. 10.1126/science.285.5432.1393 10464096

[B53] Ursini F. Maiorino M. Valente M. Ferri L. Gregolin C. (1982). Purification from Pig Liver of a Protein Which Protects Liposomes and Biomembranes from Peroxidative Degradation and Exhibits Glutathione Peroxidase Activity on Phosphatidylcholine Hydroperoxides. Biochimica Biophysica Acta (BBA) - Lipids Lipid Metabolism 710 (2), 197–211. 10.1016/0005-2760(82)90150-3 7066358

[B54] Vernet P. Aitken R. J. Drevet J. R. (2004). Antioxidant Strategies in the Epididymis. Mol. Cell. Endocrinol. 216 (1-2), 31–39. 10.1016/j.mce.2003.10.069 15109742

[B55] Vučković A. M. Bosello Travain V. Bordin L. Cozza G. Miotto G. Rossetto M. (2020). Inactivation of the Glutathione Peroxidase GPx4 by the Ferroptosis‐inducing Molecule RSL3 Requires the Adaptor Protein 14‐3‐3ε. FEBS Lett. 594 (4), 611–624. 10.1002/1873-3468.13631 31581313

[B56] Wagner S. A. Beli P. Weinert B. T. Nielsen M. L. Cox J. Mann M. (2011). A Proteome-wide, Quantitative Survey of *In Vivo* Ubiquitylation Sites Reveals Widespread Regulatory Roles. Mol. Cell. Proteomics 10 (10), M111. 10.1074/mcp.M111.013284 PMC3205876 21890473

[B57] Wang G. Wu Y. Zhou T. Guo Y. Zheng B. Wang J. (2013). Mapping of the N-Linked Glycoproteome of Human Spermatozoa. J. Proteome Res. 12 (12), 5750–5759. 10.1021/pr400753f 24191733

[B58] Wei X. Yi X. Zhu X.-H. Jiang D.-S. (2020). Posttranslational Modifications in Ferroptosis. Oxidative Med. Cell. Longev. 2020, 1–12. 10.1155/2020/8832043 PMC7718049 33294126

[B59] Weïwer M. Bittker J. A. Lewis T. A. Shimada K. Yang W. S. MacPherson L. (2012). Development of Small-Molecule Probes that Selectively Kill Cells Induced to Express Mutant RAS. Bioorg. Med. Chem. Lett. 22 (4), 1822–1826. 10.1016/j.bmcl.2011.09.047 22297109 PMC3528973

[B60] Yang L. Chen X. Yang Q. Chen J. Huang Q. Yao L. (2020). Broad Spectrum Deubiquitinase Inhibition Induces Both Apoptosis and Ferroptosis in Cancer Cells. Front. Oncol. 10, 949. 10.3389/fonc.2020.00949 32596160 PMC7304060

[B61] Yang S. H. Chang S. Y. Andres D. A. Spielmann H. P. Young S. G. Fong L. G. (2010). Assessing the Efficacy of Protein Farnesyltransferase Inhibitors in Mouse Models of Progeria. J. Lipid Res. 51 (2), 400–405. 10.1194/jlr.M002808 19965595 PMC2803242

[B62] Yang W. S. SriRamaratnam R. Welsch M. E. Shimada K. Skouta R. Viswanathan V. S. (2014). Regulation of Ferroptotic Cancer Cell Death by GPX4. Cell 156 (1-2), 317–331. 10.1016/j.cell.2013.12.010 24439385 PMC4076414

[B63] Yang W. S. Stockwell B. R. (2008). Synthetic Lethal Screening Identifies Compounds Activating Iron-dependent, Nonapoptotic Cell Death in Oncogenic-RAS-Harboring Cancer Cells. Chem. Biol. 15 (3), 234–245. 10.1016/j.chembiol.2008.02.010 18355723 PMC2683762

[B64] Young S. G. Fong L. G. Michaelis S. (2005). Thematic Review Series: Lipid Posttranslational Modifications. Prelamin A, Zmpste24, Misshapen Cell Nuclei, and Progeria-New Evidence Suggesting that Protein Farnesylation Could Be Important for Disease Pathogenesis. J. Lipid Res. 46 (12), 2531–2558. 10.1194/jlr.R500011-JLR200 16207929

[B65] Yu Y. Xie Y. Cao L. Yang L. Yang M. Lotze M. T. (2015). The Ferroptosis Inducer Erastin Enhances Sensitivity of Acute Myeloid Leukemia Cells to Chemotherapeutic Agents. Mol. Cell. Oncol. 2 (4), e1054549. 10.1080/23723556.2015.1054549 27308510 PMC4905356

[B66] Zhang J. Qiu Q. Wang H. Chen C. Luo D. (2021a). TRIM46 Contributes to High Glucose-Induced Ferroptosis and Cell Growth Inhibition in Human Retinal Capillary Endothelial Cells by Facilitating GPX4 Ubiquitination. Exp. Cell Res. 407 (2), 112800. 10.1016/j.yexcr.2021.112800 34487731

[B67] Zhang Y. Swanda R. V. Nie L. Liu X. Wang C. Lee H. (2021b). mTORC1 couples cyst(e)ine availability with GPX4 protein synthesis and ferroptosis regulation. Nat. Commun. 12 (1), 1589. 10.1038/s41467-021-21841-w 33707434 PMC7952727

[B68] Zhang Y. Tan H. Daniels J. D. Zandkarimi F. Liu H. Brown L. M. (2019). Imidazole Ketone Erastin Induces Ferroptosis and Slows Tumor Growth in a Mouse Lymphoma Model. Cell Chem. Biol. 26 (5), 623–633. 10.1016/j.chembiol.2019.01.008 30799221 PMC6525071

